# The Synergism between DHODH Inhibitors and Dipyridamole Leads to Metabolic Lethality in Acute Myeloid Leukemia

**DOI:** 10.3390/cancers13051003

**Published:** 2021-02-28

**Authors:** Valentina Gaidano, Mohammad Houshmand, Nicoletta Vitale, Giovanna Carrà, Alessandro Morotti, Valerio Tenace, Stefania Rapelli, Stefano Sainas, Agnese Chiara Pippione, Marta Giorgis, Donatella Boschi, Marco Lucio Lolli, Daniela Cilloni, Alessandro Cignetti, Giuseppe Saglio, Paola Circosta

**Affiliations:** 1 Department of Clinical and Biological Sciences, University of Turin, Orbassano, 10043 Turin, Italy; mohammad.houshmand@unito.it (M.H.); giovanna.carra@unito.it (G.C.); alessandro.morotti@unito.it (A.M.); daniela.cilloni@unito.it (D.C.); giuseppe.saglio@unito.it (G.S.); paola.circosta@unito.it (P.C.); 2 Division of Hematology, A.O. SS Antonio e Biagio e Cesare Arrigo, 15121 Alessandria, Italy; 3 Molecular Biotechnology Center, University of Turin, 10126 Turin, Italy; nicoletta.vitale@unito.it; 4 Department of Medical Sciences, University of Turin, 10124 Turin, Italy; 5 Department of Electrical and Computer Engineering, University of Utah, Salt Lake City, UT 84112, USA; valerio.tenace@utah.edu; 6 Department of Life Sciences and System Biology, University of Turin, 10124 Turin, Italy; stefania.rapelli@unito.it; 7 Department of Drug Science and Technology, University of Turin, 10124 Turin, Italy; stefano.sainas@unito.it (S.S.); agnesechiara.pippione@unito.it (A.C.P.); marta.giorgis@unito.it (M.G.); donatella.boschi@unito.it (D.B.); marco.lolli@unito.it (M.L.L.); 8 University Division of Hematology and Cell Therapy, A.O. Ordine Mauriziano, University of Turin, 10128 Turin, Italy; alessandro.cignetti@unito.it

**Keywords:** DHODH, dipyridamole, acute myeloid leukemia, apoptosis, differentiation, pyrimidine depletion, cancer metabolism

## Abstract

**Simple Summary:**

In this study, we investigated and boosted the pro-apoptotic and pro-differentiating activity of MEDS433 in acute myeloid leukemia (AML). MEDS433 is an inhibitor of Dihydroorotate Dehydrogenase, a fundamental enzyme in the *de novo* pyrimidine biosynthesis. We discovered that MEDS433 alone and in combination with classical antileukemic agents had a good apoptotic activity, but it could be reduced in vivo due to the physiological presence of uridine. On the contrary, the combination of MEDS433 and dipyridamole, a blocker of the pyrimidine salvage pathway, induced metabolic lethality and myeloid differentiation in all our AML models, while being characterized by a limited toxicity on non-AML cells.

**Abstract:**

Dihydroorotate Dehydrogenase (DHODH) is a key enzyme of the *de novo* pyrimidine biosynthesis, whose inhibition can induce differentiation and apoptosis in acute myeloid leukemia (AML). DHODH inhibitors had shown promising in vitro and in vivo activity on solid tumors, but their effectiveness was not confirmed in clinical trials, probably because cancer cells exploited the pyrimidine salvage pathway to survive. Here, we investigated the antileukemic activity of MEDS433, the DHODH inhibitor developed by our group, against AML. Learning from previous failures, we mimicked human conditions (performing experiments in the presence of physiological uridine plasma levels) and looked for synergic combinations to boost apoptosis, including classical antileukemic drugs and dipyridamole, a blocker of the pyrimidine salvage pathway. MEDS433 induced apoptosis in multiple AML cell lines, not only as a consequence of differentiation, but also directly. Its combination with antileukemic agents further increased the apoptotic rate, but when experiments were performed in the presence of physiological uridine concentrations, results were less impressive. Conversely, the combination of MEDS433 with dipyridamole induced metabolic lethality and differentiation in all AML cell lines; this extraordinary synergism was confirmed on AML primary cells with different genetic backgrounds and was unaffected by physiological uridine concentrations, predicting *in human* activity.

## 1. Introduction

The inhibition of Dihydroorotate Dehydrogenase (DHODH) has recently been found to induce differentiation in several models of acute myeloid leukemia (AML) [1]. DHODH is a mitochondrial enzyme which catalyzes the conversion of dihydroorotate to orotate, being both a fundamental enzyme in the *de novo* pyrimidine biosynthesis and in the electron transport chain [2]. However, evidence [3] suggests that DHODH inhibition acts through pyrimidine starvation, rather than cellular respiration impairment; the counter-proof is that high levels of uridine, a downstream product of DHODH in the pyrimidine biosynthesis, are able to abolish the effect of DHODH inhibitors on AML cells [1].

From this seminal discovery, several academic and industrial research groups, including ours, have designed new and more potent DHODH inhibitors, confirming original results and extending the knowledge about this topic [4,5,6,7,8,9,10]. While the exact mechanisms triggered by DHODH inhibitors on leukemic cells have not been fully elucidated [11], transcriptome analyses revealed that leukemic cells treated with DHODH inhibitors upregulate genes related to apoptosis and differentiation, and downregulate protein translation-related genes, impairing protein synthesis [6,7]. Unlike differentiation, DHODH inhibition-induced apoptosis has not been thoroughly investigated; it has probably been considered a consequence of differentiation, somehow similarly to the effect induced by all-trans retinoic acid (ATRA) in promyelocytic leukemia (APL). However, DHODH inhibitors had been previously shown to reduce the growth of solid tumors in vitro, to increase p53 levels, and to synergize with an inhibitor of p53 degradation [12]. Indeed, pyrimidines are crucial for the proliferation of living entities, and the depletion of the intracellular pyrimidine pool results in cell cycle arrest in S-phase [12].

In spite of the underlying mechanisms, DHODH inhibition is an extremely interesting strategy, as (i) it represents a new pathway to treat AML; (ii) differentiation induction has yielded incredible results in APL, probably reducing the relapse rate by working on leukemic stem cells [13], and (iii) the toxicity seems to be limited, potentially allowing DHODH inhibitors to be combined with other drugs. Not surprisingly, four different agents are currently being tested in clinical trials for AML, including old (brequinar) and new compounds (PTC299, ASLAN003, BAY2402234).

Despite this justified enthusiasm, there are also a few concerns about the efficacy of DHODH inhibitors, especially if they are used alone.

DHODH inhibitors have already failed to prove their efficacy in clinical trials on solid tumors [14,15,16], probably due to an incomplete pyrimidine starvation. In fact, cells can acquire pyrimidines from two sources: the *de novo* pathway, where DHODH plays a crucial role, and the salvage mechanism, where uridine and cytidine are acquired from the extracellular space. Resting cells generally recycle pyrimidines through the latter, while rapidly growing cells preferentially use the *de novo* pathway [17]. The salvage mechanism probably justifies the low toxicity of DHODH inhibitors in non-proliferating cells; on the other hand, it represents a natural escape that cancer cells can leverage to survive; uridine, indeed, is naturally present in plasma and can enter the cells through Equilibrative Nucleoside Transporters (hENT1/2) or, to a lesser extent, Concentrative Nucleoside Transporters (hCNT) channels.

Further concerns derive from the notion that AML biology is characterized by the subsequent emergence of multiple resistant subclones; for this reason, AML is generally treated with multidrug therapies, whether chemotherapy-based (polychemotherapy) or non-chemotherapy-based (e.g., hypomethylating agents + bcl2 inhibitors).

In order to improve DHODH inhibitors’ performances and look for synergic combinations, we decided to test multiple reasoned drug combinations in vitro. We started with Ara-C and decitabine, two pyrimidine analogues which are routinely used in AML therapy; by depleting the intracellular pyrimidine pool, in fact, MEDS433 could potentially promote the DNA incorporation of the pyrimidine analogues. Subsequently we tested an anthracycline, idarubicin; besides being part of the “3 + 7” chemotherapeutic regimen, anthracyclines can induce cell death through several mechanisms, including free radical formations, p53 and caspase activation, which are also influenced by DHODH inhibitors [12,18,19,20,21]. Finally, we hypothesized that the contemporary blockade of both the *de novo* and the salvage pyrimidine pathways could be fatal to cancer cells, which are dependent on pyrimidine supply. In particular, we associated MEDS433, the DHODH inhibitor developed by our group, to dipyridamole, an antiplatelet drug with vasodilating properties which is also known to inhibit hENT1 and hENT2 channels, thereby greatly reducing the nucleoside/nucleotide influx of the salvage pathway [22].

In summary, this work aims to: (i) characterize the antileukemic activity of DHODH inhibitors, analyzing the effect of MEDS433 on different AML cells and in different conditions, and (ii) find synergic combinations to boost the activity of DHODH inhibitors.

## 2. Results

### 2.1. MEDS433 Induces Apoptosis in Several AML Cell Lines

MEDS433 is a new, potent DHODH inhibitor, developed and characterized by our group, which can induce differentiation in multiple AML cell lines, at a 1-log lower concentration compared to brequinar [4].

**Figure 1:**
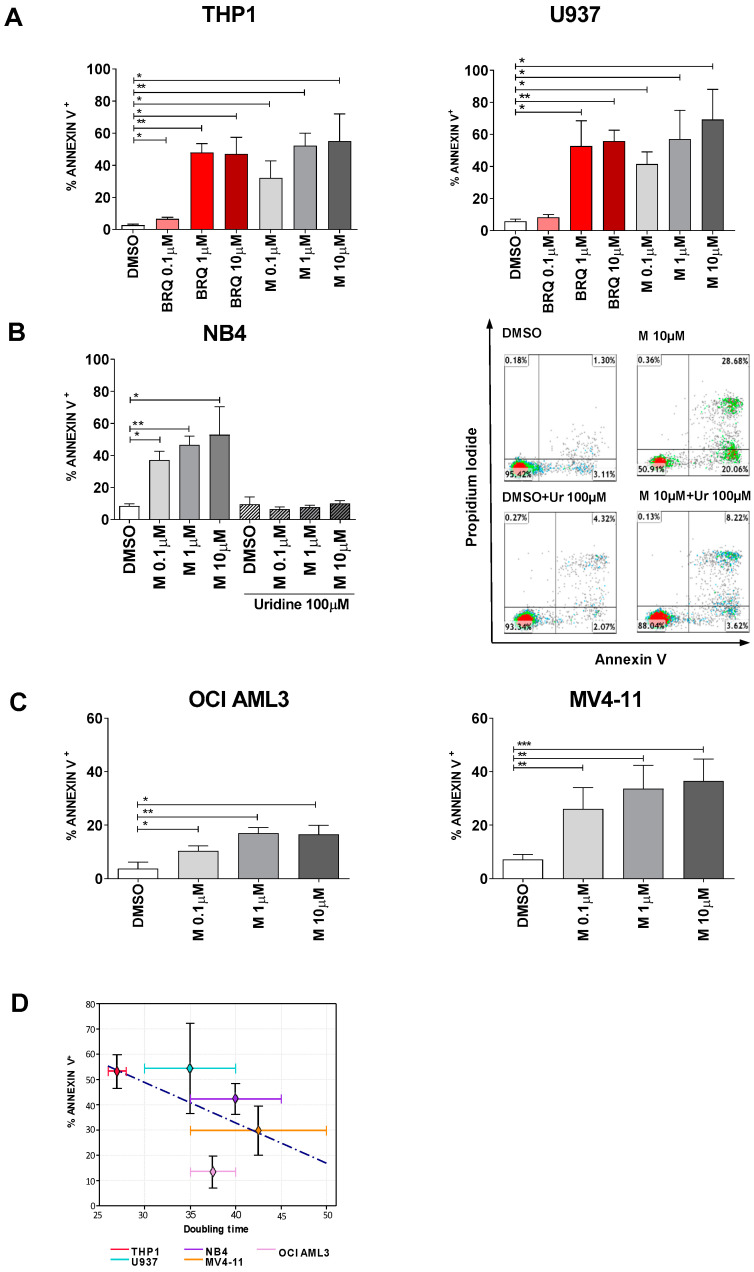
MEDS433 induces apoptosis in several acute myeloid leukemia (AML) cell lines (I). (**A**) Comparison between the apoptotic rate induced by MEDS433 and brequinar at different concentrations (*n* = 3). (**B**) Hyperphysiological levels of uridine (100 µM) abrogate the apoptotic effect of MEDS433 (*n* = 3). Right panel: Flow cytometry plots of a representative experiment of NB4 cells treated with MEDS433 ± uridine. (**C**) OCI AML3 (*n* = 4) and MV4-11 (*n* = 5) have a reduced sensitivity to MEDS433 compared to THP1, U937 and NB4 cell lines. (**D**) Regression line correlating the apoptotic rate induced by MEDS433 and the doubling time of tested AML cell lines. Apoptosis was evaluated after 3 days of treatment through Annexin V expression. DMSO indicates cells treated with dimethyl sulfoxide only. M: MEDS433. BRQ: brequinar. Ur: uridine. Statistical significance: *t*-test, * *p* < 0.05; ** *p* < 0.01; *** *p* < 0.001.

**Figure 2:**
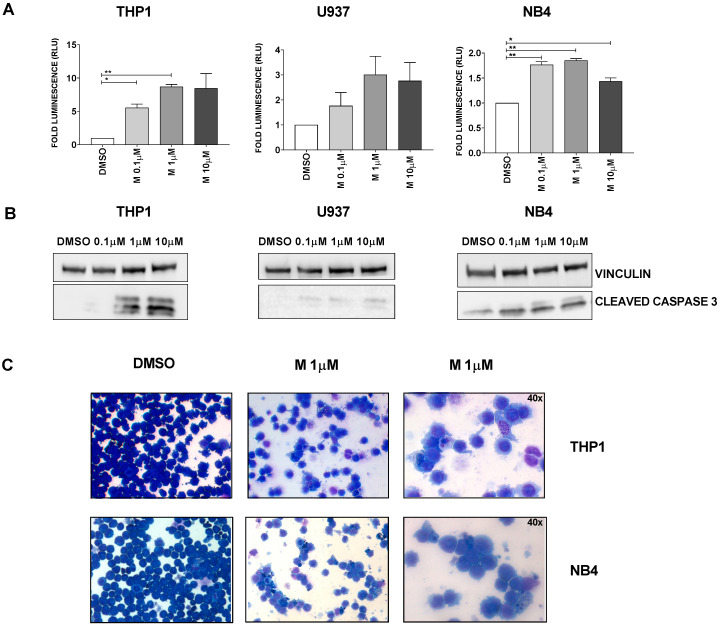
MEDS433 induces apoptosis in several AML cell lines (II). (**A**) MEDS433 treatment increases the activity of caspases 3/7, as evaluated through a specific functional assay (*n* = 3). (**B**) MEDS433 increases the levels of cleaved caspase 3 (*n* = 3). (**C**) Morphologic evaluation (Wright–Giemsa staining) of untreated (left panel) and treated cells (middle and right panels, *n* = 3). Untreated cells form a conspicuous, monomorphic population, with strongly basophilic appearance. After treatment with MEDS433, the population is significantly reduced; cells are extremely heterogeneous, with both signs of apoptosis (cytoplasmic vacuolation, karyopyknosis and karyorrhexis) and differentiation (decreased nuclear-cytoplasmic ratio and enlightening of the cytoplasm). If not otherwise specified, the magnification is 20×. Analyses were performed after 3 days of treatment. DMSO: dimethyl sulfoxide only. Statistical significance: *t*-test, * *p* < 0.05; ** *p* < 0.01.

Here we show that MEDS433 also has a strong pro-apoptotic effect on several AML cell lines, as demonstrated by: (i) the Annexin V assay (Figure 1); (ii) the increased caspase-3/7 activity (Figure 2A) and the increased levels of cleaved caspase 3 (Figure 2B and Figure S8) after exposure to MEDS433; (iii) the reduction in apoptosis when experiments were performed in the presence of a caspase inhibitor (Z-VAD-fmk, data not shown); and (iv) the morphologic evaluation of cells treated with MEDS433 (Figure 2C).

The pro-apoptotic and the pro-differentiating activities of MEDS433 share several similarities [4]: (i) MEDS433 could induce apoptosis at a 1-log lower concentration compared to brequinar (Figure 1A); (ii) the apoptotic effect was totally abrogated when uridine was added at hyperphysiological concentrations (100 µM), in all tested cell lines (Figure 1B, Figure 8A and Figure S6), indicating that apoptosis was indeed due to pyrimidine starvation rather than off-target effects, and (iii) both MEDS433 pro-apoptotic and pro-differentiating activities were conserved in niche-like conditions, i.e., when AML cell lines were co-cultured with a stromal cell line or in hypoxic conditions (Figure S1C,D).

While MEDS433 could induce apoptosis (Figure 1) and a dramatic drop in the number of viable cells (Figure S1) on multiple AML cell lines with different genetic backgrounds (Table S1), Figure 1 clearly shows that different cell lines have different sensitivity to DHODH inhibition. In particular, MEDS433 was more active on THP1, U937 and NB4, while on OCI AML3 and MV4-11 the results were less impressive. There are probably multiple factors responsible for this heterogeneity. One hypothesis is that low proliferating cells could be less sensitive to DHODH inhibitors, as they could still rely on the salvage pathway for the pyrimidine supply. Accordingly, Figure 1D suggests that the apoptotic rate correlates with the doubling time of the cell lines (r^2^ = 0.3), confirming previous findings [23]. Different genetic backgrounds could also influence the sensitivity to DHODH inhibitors; for example, U937, THP1 and NB4 cells, i.e., the most sensitive cell lines to MEDS433, are all p53 mutated. Disappointingly, we did not observe a significant increase in p53 expression in these cell lines upon treatment, while we confirmed the upregulation of wild type p53 in OCI AML3 and MV4-11, in accordance with the literature (Figure S2) [12].

**Figure 3:**
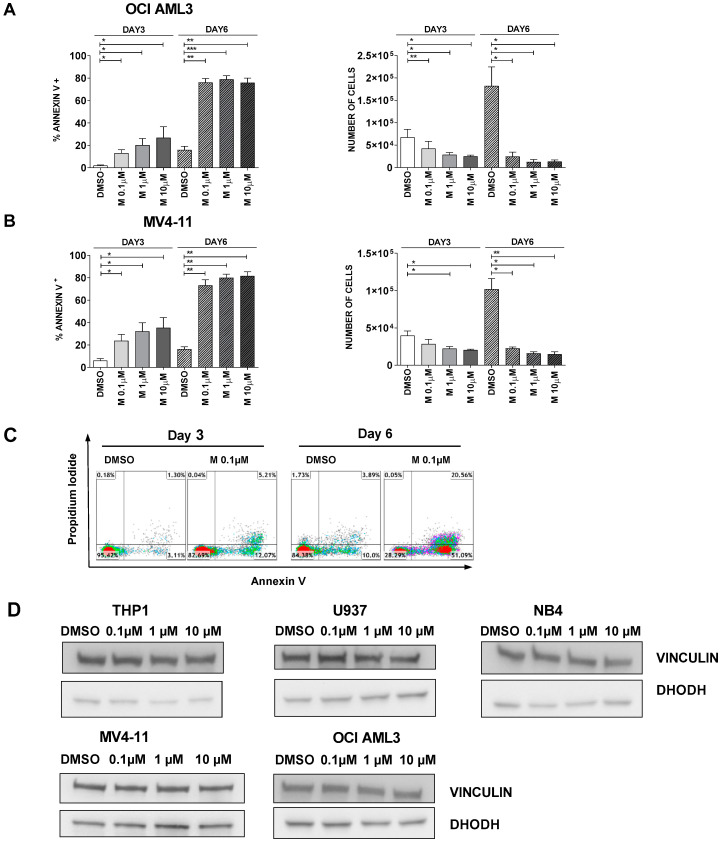
MEDS433 becomes highly effective also in partially resistant cell lines if the time of exposure is increased. (**A**,**B**) Apoptotic rate and corresponding viable cell counts of OCI AML3 (**A**, *n* = 3) and MV4-11 (**B**, *n* = 3) treated for 3 or 6 days with MEDS433 at different concentrations. (**C**) Flow cytometry plots of a representative experiment of MV4-11 cells treated with MEDS433 for 3 or 6 days. (**D**) Dihydroorotate Dehydrogenase (DHODH) protein levels before and after treatment with MEDS433 at different concentrations. (*n* = 3) DMSO: dimethyl sulfoxide. M: MEDS433. Statistical significance: *t*-test, * *p* < 0.05; ** *p* < 0.01; *** *p* < 0.001.

Beyond the reasons underlying different responses to DHODH inhibition, a prolonged exposure to MEDS433 could rescue partially resistant cell lines. In particular, when OCI-AML3 and MV4-11 cells were treated for 6 instead of 3 days, apoptosis was significantly increased, and the number of viable cells was remarkably reduced compared to the control (Figure 3A–C). Figure 3D and Figure S9 show that treatment with MEDS433 does not influence the levels of DHODH expression; this is in accordance with the literature [24] and suggests that MEDS433 could be administered for a prolonged period of time, maintaining its effectiveness.

### 2.2. Apoptosis Is Both the Result of Differentiation and a Direct Effect of DHODH Inhibition

**Figure 4:**
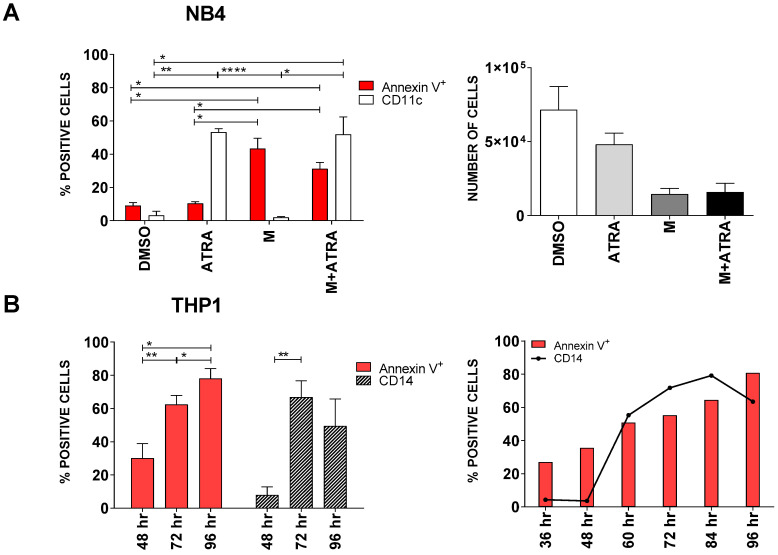
MEDS433 can induce apoptosis and differentiation separately. (**A**) Left panel: apoptotic and differentiating rate of NB4 cells treated with ATRA, MEDS433 or both (*n* = 3). Right panel: viable NB4 cells after treatment with ATRA, MEDS433 or both (*n* = 3). (**B**) Kinetic of apoptosis and differentiation of THP1 cells treated with MEDS433. In all experiments, MEDS433 was utilized at 1 µM and ATRA at 0.1 µM. Differentiation of NB4 cells was best evaluated with CD11c expression, while THP1 cells with CD14 expression. The analyses shown in panel A were performed after 3 days of treatment. DMSO: dimethyl sulfoxide. ATRA: all-trans retinoic acid. M: MEDS433. hr: hours. (**A**) Statistical significance: Anova/Tukey, * *p* < 0.05; ** *p* < 0.01; **** *p* < 0.0001. (**B**) Statistical significance: *t*-test, * *p* < 0.05; ** *p* < 0.01.

Working on NB4 cells, a promyelocytic cell line, we observed for the first time that differentiation and apoptosis were partially independent effects of DHODH inhibition. Figure 4A shows that, after 3 days of treatment, ATRA induced a strong expression of CD11c on leukemic cells (53.33% ± 2.02) while annexin expression was still minimal (10.4% ± 0.94), indicating that cells were differentiating but not dying yet. On the contrary, MEDS433 treatment greatly increased the percentage of apoptotic cells (43.43% ± 6.22), but it did not cause differentiation (1.97% ± 0.48). The combination of MEDS433 and ATRA induced differentiation (51.89% ± 10.53) and apoptosis (31.3% ± 3.79) simultaneously. Accordingly, the number of viable cells was modestly reduced when NB4 cells were just treated with ATRA, while it notably declined when cells were treated with MEDS433 alone or in combination with ATRA (Figure 4A, right panel).

In all other tested cell lines, MEDS433 induced both apoptosis and differentiation; however, by analyzing the kinetics of these two phenomena, we noticed that the expression of annexin V anticipated the increase in the differentiation markers (Figure 4B and Figure S3). These results indicate that at least a subset of cells undergoing apoptosis are not at the end of their differentiation process.

DHODH inhibitors, hence, work differently from pure differentiating agents, as they are able to directly cause apoptosis.

### 2.3. The Combination of MEDS433 with Classical Antileukemic Agents Results in Near-Additive Effects

**Figure 5:**
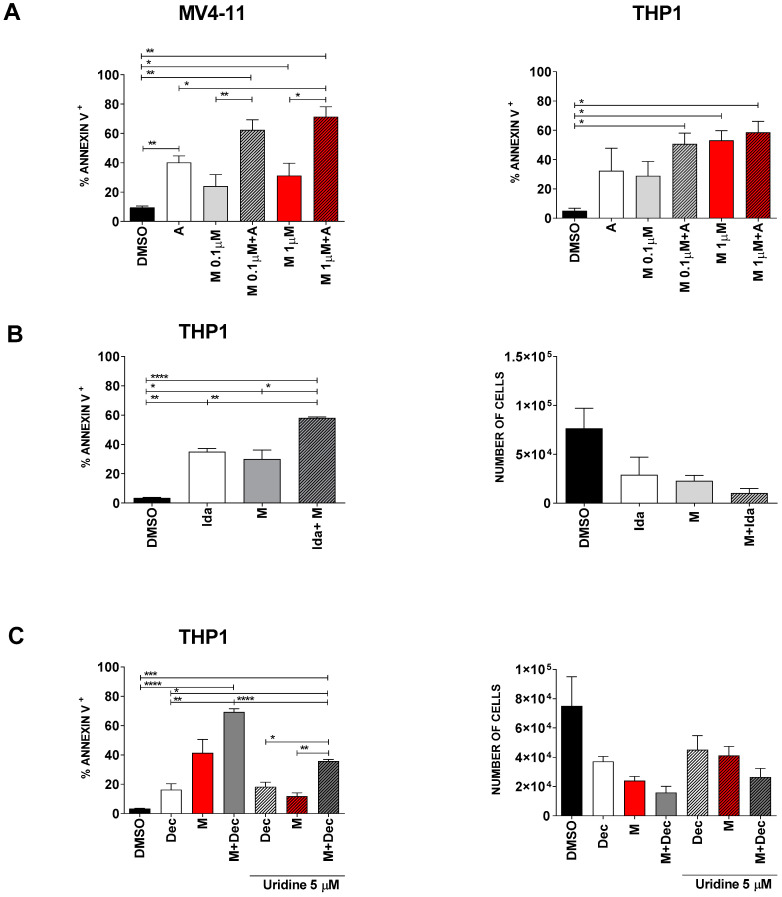
The combination of MEDS433 with classical antileukemic agents results in near-additive effects. (**A**) Apoptosis induced by MEDS433, Ara-C and their combination on MV4-11 (left panel, *n* = 4) and THP1 (right panel, *n* = 3) cells. (**B**) Apoptotic rate (left panel) and cell viability (right panel) of THP1 cells treated with MEDS433, idarubicin or both (*n* = 3). (**C**) Apoptotic rate (left panel, *n* = 3) and cell viability (right panel, *n* = 3) of THP1 cells treated with decitabine, MEDS433 or both, with or without physiological levels of uridine (5 µM). If not otherwise specified, MEDS433 was utilized at 0.1 µM. Apoptosis and cell counts were evaluated after 3 days of treatment. DMSO: dimethyl sulfoxide. M: MEDS433. A: Ara-C. Ida: idarubicin. Dec: decitabine. Statistical significance: Anova/Tukey, * *p* < 0.05; ** *p* < 0.01; *** *p* < 0.001; **** *p* < 0.0001.

In order to increase the apoptotic rate of AML cell lines, we first combined our DHODH inhibitor with some selected chemotherapeutic agents that are classically used to treat AML, i.e., Ara-C, anthracyclines and decitabine. The concentrations of Ara-C (1 µM), idarubicin (0.005 µM) and decitabine (0.250 µM) were chosen to induce a 15 to 40% apoptotic rate when administered alone, in order to explore synergistic combinations. Figure 5 and Figure S4 show that the combination of MEDS433 with Ara-C, idarubicin or decitabine significantly increases the apoptotic rate of either compound alone, resulting in a near-additive effect. However, in order to mimic in vivo conditions, we performed these experiments also in the presence of low, physiological, uridine levels. Figure 5C shows that even low levels of uridine reduce the ability of MEDS433 to induce apoptosis, resulting in a substantial decrease in efficacy of MEDS433 alone and in combination with decitabine (from 69.32% ± 2.13 to 35.80% ± 1.13).

### 2.4. The Combination of MEDS433 with hENT1/2 Inhibitors Results in Synergistic Effects Against AML

**Figure 6:**
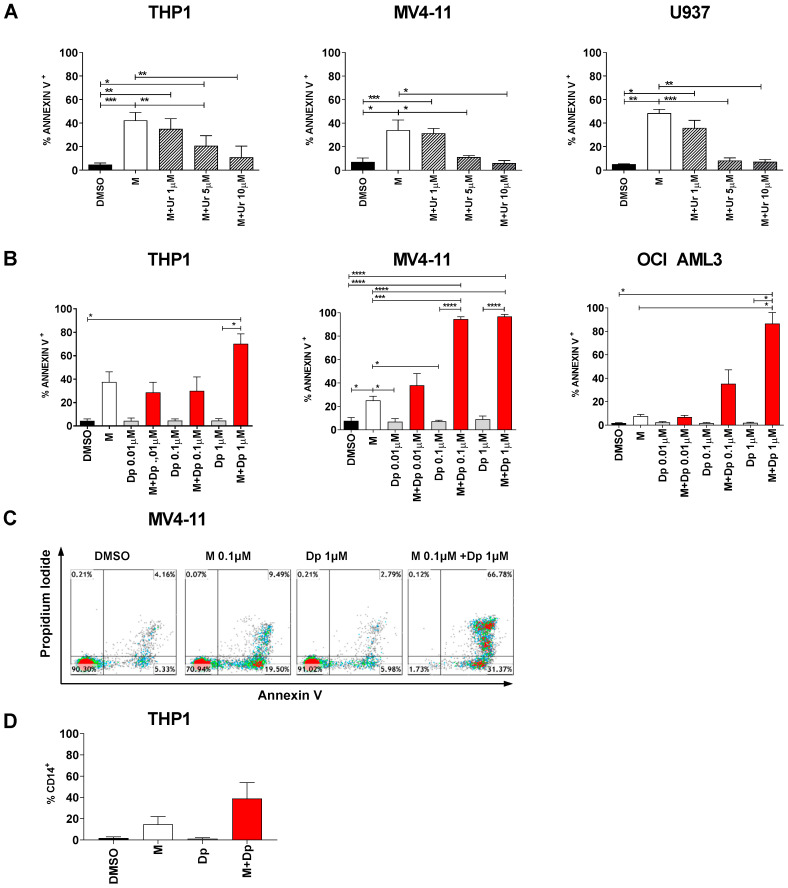
The combination of MEDS433 and dipyridamole results in synergistic effects. (**A**) Analysis of the apoptotic rate induced by MEDS433 on THP1 (*n* = 4), MV4-11 (*n* = 3) and OCI-AML3 cells (*n* = 3), when utilized alone or in the presence of uridine at low concentrations (1 to 10 µM). (**B**) Apoptosis induced by MEDS433, dipyridamole and their combination on THP1 (*n* = 3), MV4-11 (*n* = 3) and OCI-AML3 cells (*n* = 3). (**C**) Flow cytometry plots on a representative experiment on MV4-11 cells treated with MEDS433, dipyridamole and their combination (**D**) Differentiation induced by MEDS433 and dipyridamole, alone or in combination, on THP1 cells; the differentiation analysis was performed on day 2 (*n* = 3). In all experiments, MEDS433 was utilized at 0.1 µM and apoptosis was evaluated after 3 days of treatment. DMSO: dimethyl sulfoxide. M: MEDS433. Dp: dipyridamole. Statistical significance: Anova/Tukey, * *p* < 0.05; ** *p* < 0.01; *** *p* < 0.001; **** *p* < 0.0001.

Faced with results reported in Figure 5C, we decided to investigate the antagonist role of uridine on DHODH inhibitors. Physiological uridine plasma levels are variable, approximately ranging from 2 to 6 µM, being influenced by many factors, from physical activity to beer consumption [25,26]. Figure 6A shows that the apoptotic rate induced by MEDS433 was indeed reduced at increasing uridine concentrations, potentially limiting the role of DHODH inhibitors in AML in vivo.

**Figure 7:**
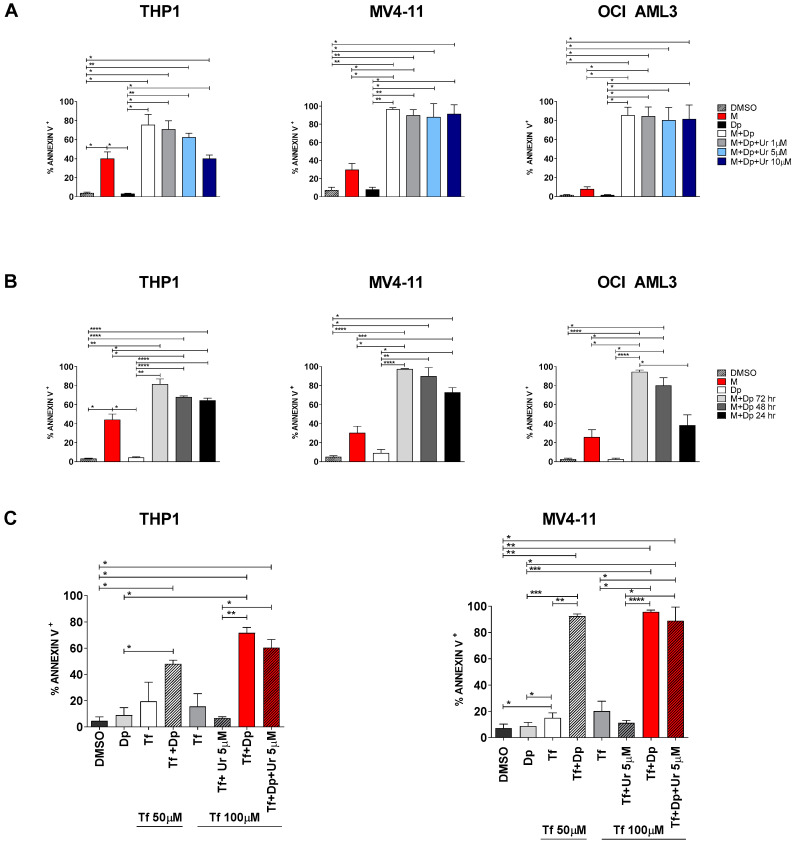
The synergism between DHODH inhibitors and dipyridamole is confirmed when in vivo conditions are mimicked. (**A**) Apoptosis induced by MEDS433 in combination with dipyridamole in the presence of uridine at low concentrations (1 to 10 µM) on THP1 (*n* = 3), MV4-11 (*n* = 3) and OCI AML3 cells (*n* = 3). (**B**) Cells were exposed to MEDS433 for 3 days and to dipyridamole for 3, 2 or 1 concomitant day(s), as shown in the figure legend; apoptosis was then evaluated on day 3 (*n* = 3). (**C**) Apoptosis induced by teriflunomide, dipyridamole and their combination, alone or in the presence of physiological uridine concentrations (5 µM), on THP1 (*n* = 3) and MV4-11 cells (*n* = 3). DMSO: dimethyl sulfoxide. M: MEDS433. Dp: dipyridamole. Ur: uridine. Tf: teriflunomide. Statistical significance: Anova/Tukey, * *p* < 0.05; ** *p* < 0.01; *** *p* < 0.001; **** *p* < 0.0001.

In order to minimize this issue, we decided to block both the *de novo* and the salvage pathway by combining MEDS433 to dipyridamole, a known inhibitor of nucleoside/nucleotide transport channels hENT1/2. As we were looking for a synergistic effect, we challenged our system using low concentrations of MEDS433 (0.1 µM) and increasing concentrations of dipyridamole. Dipyridamole alone had no effect on any AML cell line, while MEDS433 confirmed its performances in the less favorable conditions (0.1 µM). Notably, the combination of MEDS433 and dipyridamole resulted in a dramatic increase in the apoptotic rate, especially with dipyridamole concentrations above 0.1 µM. All tested cell lines, even the most resistant to DHODH inhibitors, experienced this phenomenon (Figure 6B,C and Figure S5A). Figure 6D shows that the synergy of this combination was not limited to apoptosis, but rather extended to the differentiating effect as well. Unlike other experiments, the differentiation analysis had to be performed on day 2, resulting in worse results compared to Figure S1D, because on day 3 the apoptotic rate was too high and compromised the reliability of results. We also challenged this new combination by (i) performing experiments in the presence of low uridine levels (Figure 7A and Figure S5B), and (ii) adding dipyridamole for a limited amount of time (Figure 7B): in both cases, the apoptotic rate was unaffected or largely preserved.

Finally, in order to confirm the underlying mechanism, we substituted (i) MEDS433 with teriflunomide, another DHODH inhibitor (Figure 7C), and (ii) dipyridamole with dilazep, another hENT1/2 channel blocker (Figure S5C). In both cases, the synergism between a DHODH inhibitor and a hENT1/2 blocker was confirmed. These experiments are particularly important because teriflunomide is already commercialized as an immunosuppressor and thus readily available; however, being 320-times less potent than MEDS433, teriflunomide alone did not have a remarkable apoptotic effect in any AML cell line, even at high doses; on the contrary, its combination with dipyridamole resulted in the net increase in the apoptotic rate far above 60% in every tested AML cell line, even when in vivo conditions were mimicked (uridine 5 µM).

### 2.5. Uridine Reversal: A Matter of Concentration and Transporters

**Figure 8:**
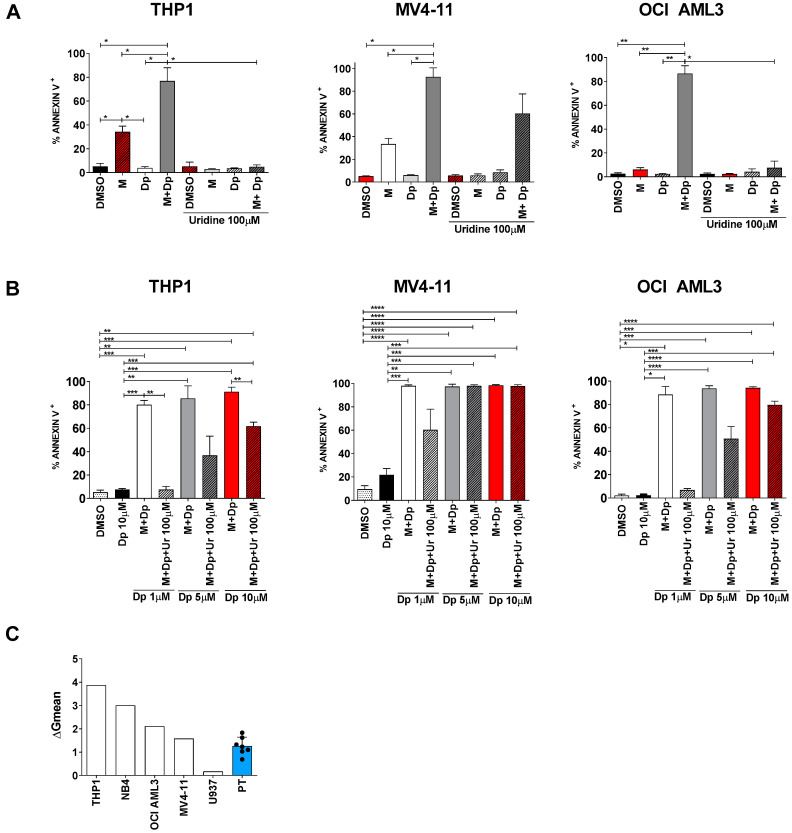
The uridine rescue is influenced by uridine concentrations and by the availability of hENT2 transporters. (**A**) Apoptosis induced by MEDS433, dipyridamole or their combination, with and without uridine at 100 µM, on THP1 (left panel, *n* = 3), MV4-11 (middle panel, *n* = 3) and OCI AML3 cells (right panel, *n* = 3). Dipyridamole was utilized at the standard dose of 1 µM. (**B**) Apoptosis induced by MEDS433 in combination with increasing dipyridamole concentrations, with and without uridine at 100 µM on THP1 (left panel, *n* = 4), MV4-11 (middle panel, *n* = 4) and OCI AML3 cells (right panel, *n* = 3). (**C**) hENT2 expression levels in utilized cell lines and primary AML cells, assessed by flow cytometry. In all experiments, MEDS433 was utilized at 0.1 µM and apoptosis was evaluated after 3 days of treatment. DMSO: dimethyl sulfoxide. M: MEDS433. Dp: dipyridamole. Ur: uridine. PT: patients. ΔGmean: refers to Section 4.6. Statistical significance: Anova/Tukey, * *p* < 0.05; ** *p* < 0.01; *** *p* < 0.001, **** *p* < 0.0001.

When uridine was added to the combination experiments (MEDS433 + dipyridamole) at hyperphysiological concentrations (100 µM), apoptosis was almost totally abrogated in THP1, OCI AML3 and NB4 cells, and reduced in MV4-11 and U937 cells (Figure 8A and Figure S6); similar results were obtained when dipyridamole was substituted with dilazep (Figure S5C). These data confirm that the combination induces apoptosis through pyrimidine starvation rather than off-target effects, but they also demonstrate that low dose dipyridamole is not able to fully block the salvage pathway. However, even at the oversaturated concentration of uridine (100 µM), high dipyridamole concentrations (10 µM) could restore almost totally the anti-leukemic activity of the combination treatment, probably by inhibiting the vast majority of pyrimidine access channels (Figure 8B). Similar results were obtained with dilazep (Figure S5C). The reason why different cell lines have different sensitivity to the combination therapy, are differently rescued by high uridine concentrations and react differently to high dipyridamole concentrations is not clear; one hypothesis is that the sensitivity to pyrimidine depletion could inversely correlate with the levels of hENT2 protein, as suggested by expression data shown in Figure 8C.

### 2.6. Approaching the Clinical Setting (I): Effectiveness

**Figure 9:**
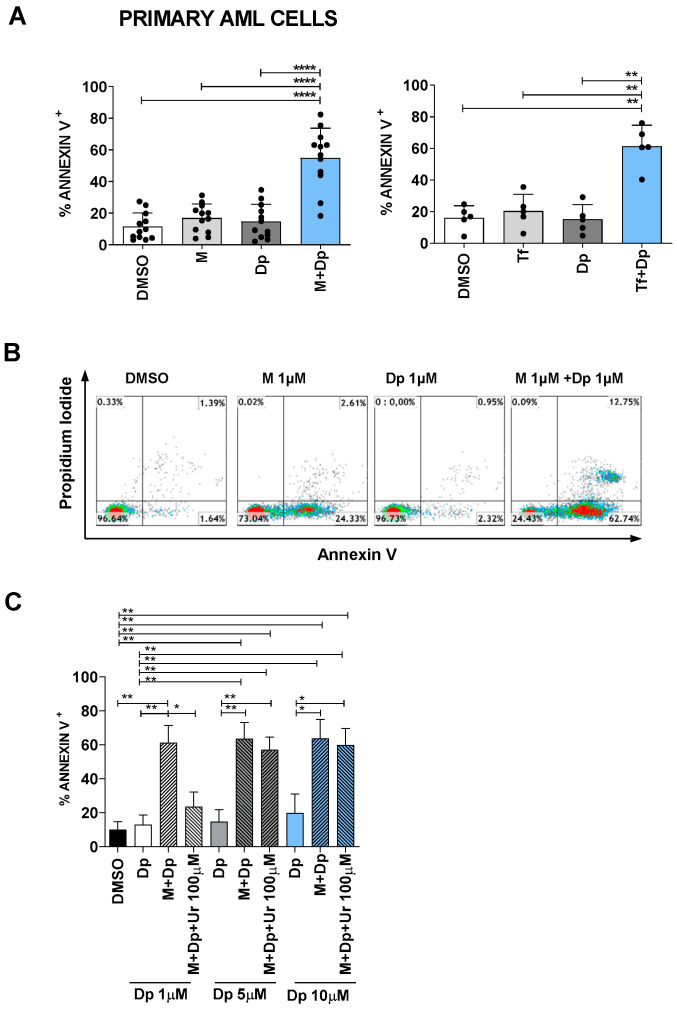
The combination of dipyridamole and DHODH inhibitors is effective against primary AML cells. (**A**) Apoptosis induced by MEDS433 (left panel, *n* = 12) or teriflunomide 100 µM (right panel, *n* = 5), alone and in combination with dipyridamole, on AML primary cells. (**B**) Flow cytometry plots of a representative sample of primary AML cells treated with MEDS433, dipyridamole and their combination (patient 3 of Table S2). (**C**) Apoptotic rate induced by MEDS433 alone and in combination with increasing dipyridamole concentrations, with or without uridine at hyperphysiological concentrations (100 µM), on primary AML samples (*n* = 4). In all the experiments, MEDS433 was utilized at 1 µM and apoptosis was evaluated after 3 days of treatment; unless otherwise specified, dipyridamole was utilized at 1 µM. DMSO: dimethyl sulfoxide. M: MEDS433. Dp: dipyridamole. Tf: teriflunomide. Ur: uridine. Statistical significance: Anova/Tukey, * *p* < 0.05; ** *p* < 0.01; *** *p* < 0.001, **** *p* < 0.0001.

In order to mimic more closely the human setting, we substituted AML cell lines with AML primary cells: again, while dipyridamole, MEDS433 or teriflunomide alone could not induce apoptosis, the combination of either DHODH inhibitor with dipyridamole resulted in a strong synergistic effect (Figure 9A,B). In particular, of the 12 samples tested, 10 had an average apoptotic rate of 61.49% (range 43.92–82.40%) after just 3 days of treatment; the two remaining samples showed less impressive results (apoptotic rate of 18.3% and 26.3% respectively), demonstrating to be less sensitive but not completely non-responders. When this experiment was repeated in the presence of uridine 100 µM, the apoptotic rate was largely reduced, excluding again off-target effects; however, high dipyridamole concentrations could almost totally rescue the apoptotic rate (Figure 9C). Noteworthily, all primary AML samples contained >75% blasts as detected by flow cytometry, and 10 out of 12 patients had a high-risk AML, whether for the European Leukemia Net risk score or because they had therapy-related or secondary AML (post myelodysplastic or myeloproliferative syndromes), as shown in Table S2. On the whole, primary cells showed high sensitivity to the combination therapy, behaving similarly to OCI AML3 and MV4-11 cells. Not surprisingly, their hENT2 levels were comparable (Figure 8C).

### 2.7. Approaching the Clinical Setting (II): Toxicity

**Figure 10:**
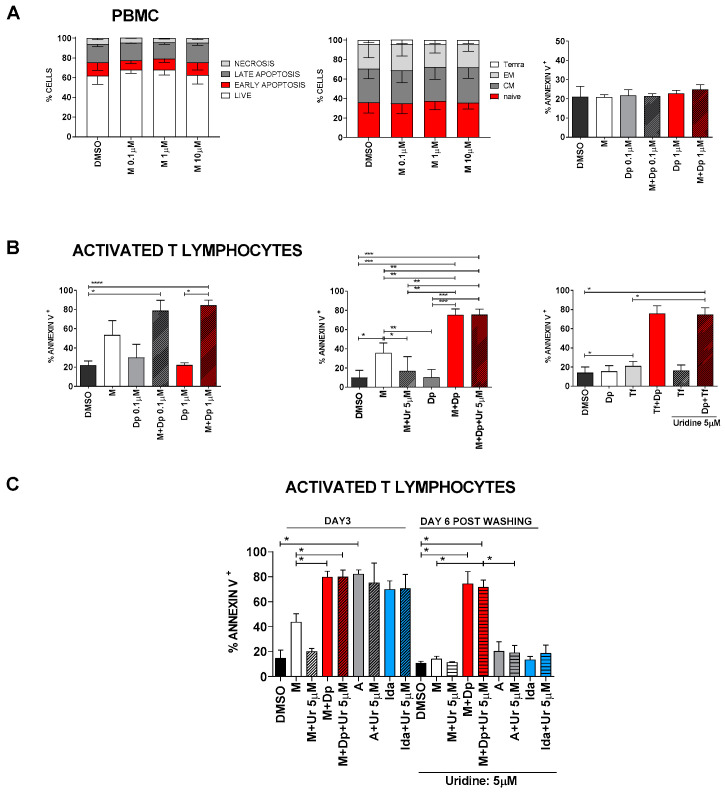
Toxicity of DHODH inhibitors alone and in combination with dipyridamole. (**A**) Left panel: apoptosis distribution induced by MEDS433 on PBMC, based on annexin V vs. propidium iodide expression (*n* = 3). Middle panel: differentiation of T-lymphocytes treated with MEDS433 at increasing concentrations (*n* = 3); the analysis was performed on the CD3+ population, identifying the following subsets: naïve (CD45RA + CD62L+), CM (central memory: CD45RA-CD62L+), EM (effector memory: CD45RA-CD62L-), and Temra (terminally differentiated effector memory CD45RA + CD62L-). Right panel: apoptosis induced by MEDS433 at 0.1 µM, dipyridamole and their combination on PBMC (*n* = 3). (**B**) Apoptosis induced by MEDS433 alone (0.1 µM) or in combination with dipyridamole on activated T-lymphocytes, without (left panel, *n* = 3) or in the presence of uridine at the physiological concentration of 5 µM (middle panel, *n* = 3). The same experiment was performed by replacing MEDS433 with Teriflunomide 100 µM (right panel, *n* = 3). (**C**) Comparison between the long and short-term toxicity of MEDS433 (0.1 µM) vs. chemotherapy (Idarubicin or Ara-c) on activated T-lymphocytes. The cells were treated for 3 days with MEDS433 alone and in combination with dipyridamole, or with Ara-C or idarubicin. After 3 days, cells were washed and replated without drugs, in the presence of physiological uridine concentrations (5 µM). If not otherwise specified, apoptosis was evaluated after 3 days of treatment. PBMC: peripheral blood mononuclear cells. DMSO: dimethyl sulfoxide. M: MEDS433. Dp: dipyridamole. Tf: teriflunomide. Ur: uridine. A: Ara-C. Ida: idarubicin. Statistical significance: Anova/Tukey, * *p* < 0.05; ** *p* < 0.01; *** *p* < 0.001, **** *p* < 0.0001.

Blocking the *de novo* pyrimidine biosynthesis and/or the salvage pathway could be theoretically toxic even for non-cancer cells. Figure 10A and Figure S7 show that neither MEDS433 alone nor its combination with dipyridamole induced apoptosis on peripheral blood mononuclear cells (PBMC) or monocytes alone. Moreover, MEDS433 was not toxic on mesenchymal cells (Figure S1C) and did not influence lymphocyte differentiation (Figure 10A, middle panel). However, when lymphocytes were activated with phytohemagglutinin (PHA)/IL2/IL7, treatment with MEDS433 induced a significant increase in the apoptotic rate, which was enhanced by dipyridamole (Figure 10B). This effect was confirmed when MEDS433 was substituted with teriflunomide and was not attenuated in the presence of physiological uridine levels (from 75.26% ± 6.2 to 75.37% ± 5.86, Figure 10B, middle and right panel). To compare the immunosuppressive potency of MEDS433 + dipyridamole and chemotherapy, we exposed activated T-lymphocytes to a short course of antileukemic treatment (MEDS433 + dipyridamole vs. Ara-C vs. idarubicin), and then observed their apoptotic rate right after the exposure (day 3) and after 6 days of recovery in the presence of uridine at physiological levels. Figure 10C shows that the combination of MEDS433 + dipyridamole has a strong immunosuppressive effect, comparable to chemotherapeutic agents, but far more sustained over time.

## 3. Discussion

The major advances of the few last years in understanding AML biology have been essential to develop new targeted drugs, such as FLT3 and IDH1/2 inhibitors. However, these “mutation-specific” drugs only address one clone, while we have learnt that AML is often an extremely heterogeneous and dynamic disease, with multiple subclones emerging or disappearing depending on their selective advantage. Therapeutic agents are a major variable influencing this selective advantage: classical chemotherapeutic drugs often select chemo-resistant clones, e.g., those harboring p53 mutation or complex karyotypes, while mutation-specific drugs, if used alone, generally induce clonal escape.

For this reason, the wise combination of multiple synergic drugs or approaches could be fundamental to efficiently target AML and avoid the emergence of resistance. Differentiating and pro-apoptotic drugs could be essential ingredients of these combinations as they could (i) force immature cells to differentiate, increasing their exposure to other drugs, and (ii) be effective on a wide variety of subclones, not being mutation specific. Not surprisingly venetoclax, a bcl-2 inhibitor with strong pro-apoptotic effects, is an emerging and extremely promising drug in the field, especially when combined with other drugs [27]. Unfortunately, all drugs with differentiating properties available today only work in the presence of specific mutations (e.g., ATRA, IDH1/2 and FLT3 inhibitors), and not ubiquitously [28,29].

In our work, we decided to focus on another non-mutation-restricted mechanism: the metabolism. It has long been known that oncogenes deviate several metabolic pathways to support cancer growth, leading to metabolic addiction [30]; on the other hand, the dysregulation of metabolism can be directly tumorigenic [31]. Since the first attempts in the 1940s [32], classical antimetabolites (anti-folate drugs, pyrimidine or purine analogues) have represented a mainstay of cancer therapy.

In 2016, a series of compounds inducing pyrimidine depletion, i.e., DHODH inhibitors, were found to promote differentiation in several AML models [1], underlying the wide influence of metabolism on cancer cells’ behavior. From that moment, the field rapidly expanded, with multiple research teams creating new agents that joined brequinar, an old but potent DHODH inhibitor, in clinical trials. Our group has recently developed MEDS433, a DHODH inhibitor which is able to induce differentiation at a 1-log lower concentration compared to brequinar [4].

Given its mechanism of action, and previous results in solid tumors, we hypothesized that DHODH inhibition could directly influence apoptosis, so we started to investigate this phenomenon in AML. Indeed, all tested AML cell lines underwent apoptosis when treated with MEDS433, with the less sensitive cell lines responding to a prolonged exposure. Apoptosis was abrogated by high dose uridine, indicating an on-target effect, and was preserved in niche-like conditions, supporting previous data showing a decrease in the number of self-renewing cells in vivo [1]. Finally, we demonstrated that apoptosis was not only the result of differentiation, but also a direct effect of DHODH inhibition.

Being able to induce both differentiation and apoptosis, and targeting a non-mutation-restricted metabolic pathway, DHODH inhibitors could represent the perfect drugs to obtain synergic treatments in AML, so we started to investigate new combinations.

Several studies in solid tumors have previously shown that DHODH inhibition could sensitize cancer cells to chemotherapy, eventually overcoming chemoresistance [33,34,35], so we started our search for synergy with Ara-C, decitabine and idarubicin. Besides being classical agents used in AML, the first two are pyrimidine analogues, whose DNA incorporation could be promoted by pyrimidine starvation, while idarubicin could potentiate the proapoptotic activity of DHODH inhibitors. In all experiments combining MEDS433 with these chemotherapeutic agents, the apoptotic rate was significantly increased, suggesting that the addition of DHODH inhibitors could improve the performances of hypomethylating agents or those of the traditional “3 + 7” induction regimen, and possibly overcome chemoresistance. Indeed, Imanishi et al. have already shown that DHODH inhibition could restore sensitivity to 5-azacytidine in resistant cell lines, in vitro [36].

However, none of these combinations resulted in synergistic effects. Moreover, despite these promising in vitro results, our major concern was the in vivo, and most importantly, the “*in human*” performance of DHODH inhibitors: in fact, despite good in vivo results on mice [37], brequinar never demonstrated a significant activity in clinical trials, at least in solid tumors [14,15,16]. There are probably multiple reasons for this phenomenon, but one of them is thought to be an incomplete pyrimidine starvation [38], possibly due to a different schedule of administration or different uridine plasma levels between humans and mice [39]. The key point of these failures, hence, could lie in the physiological presence of uridine in patients’ plasma and the possibility for cancer cells to rely on the salvage pathway for pyrimidine supply. Indeed, we demonstrated that MEDS433 showed less impressive results when uridine was added at physiological levels *in vitro,* mimicking human conditions. In order to block both the *de novo* and the salvage pathway, we combined MEDS433 to dipyridamole, a known inhibitor of nucleoside/nucleotide transport channels.

This association resulted in the dramatic increase in the apoptotic rate in all tested cell lines; besides, the synergy applied also to the differentiation process, making this combined treatment a reminiscent of ATRA and arsenic therapy in promyelocytic leukemia.

The block of both pyrimidine sources retained its effectiveness even when MEDS433 was substituted with a much less potent DHODH inhibitor, teriflunomide, which is already commercialized for multiple sclerosis. More importantly, the combination of MEDS433 with dipyridamole, but not the individual compounds, was extremely effective in inducing apoptosis in several AML primary cells: 10 out of the 12 tested samples were highly sensitive, with a median apoptotic rate exceeding 60% after just 3 days of treatment, despite being characterized for the vast majority by a high-risk profile.

Preliminary data suggest that the sensitivity to our combination therapy could correlate with the abundance of hENT2 channels, i.e., with the ability of the cells to salvage pyrimidines. Noteworthily, all primary AML cells tested had intermediate–low hENT2 levels, endorsing this hypothesis.

Although our results will need confirmatory experiments, we can already predict, based on our in vitro data, that the effect of the combined treatment on AML cells should not be affected by physiological uridine levels in vivo. Indeed, the pro-apoptotic effect of MEDS433 + dipyridamole on AML cell lines was unchanged or negligibly reduced when experiments were performed with uridine 5 μM. Moreover, we demonstrated that the combination of MEDS433 with slightly higher dipyridamole concentrations (between 1 and 5 µM) could still induce apoptosis in extreme conditions, i.e., with uridine concentrations 20–50 times more than normal. This large margin of safety represents a guarantee against possible escape mechanisms that leukemic cells could play out, such as transporter upregulation.

Given this dramatic effectiveness on several AML cells, we investigated whether this combination could be toxic also for non-cancer cells. Our data show that toxicity is limited to proliferating cells, and in particular activated T-lymphocytes, which is consistent with the use of DHODH inhibitors as immunosuppressants. This effect could reduce the ability of patients to respond to viral infections, but DHODH inhibitors are currently under investigation for their activity against RNA-virus [40]. The combination therapy could also inhibit T cells with antileukemic potential; while this phenomenon could be marginal in the presence of a high disease burden, when antileukemic T cells are generally anergized or exhausted [41], it could limit the possibility to further associate some immunotherapeutic agents (e.g., bi-specific antibodies or checkpoint inhibitors). In the post-transplant setting, our combination therapy could contemporarily target leukemia and suppress activated lymphocytes responsible for graft-versus-host disease (GVHD), representing a significant advantage over classical anti-GVHD drugs, that are just characterized by immunosuppressive properties.

In the 1990s, a limited number of studies investigated the combination of drugs inhibiting both the salvage pathway and the *de novo* synthesis in solid tumors, obtaining limited effectiveness in clinical trials [42,43]. Several hypotheses were formulated to explain this failure [38], including an incomplete inhibition of the pyrimidine supply, whether for a wrong brequinar administration schedule or because other channels allowed a minimal quantity of uridine to enter the cells. Another hypothesis is that this combination was cytostatic in cancer cells of solid tumors [38]. Our data show that this combination is highly effective in AML, where it has cytotoxic effects. As a matter of fact, the kinetics of solid and hematological tumors, and especially AML, are extremely different; moreover, the pyrimidine starvation also induces differentiation in the AML settings. Taken together these results suggest that the combination therapy of DHODH inhibitors and hENT blockers could work in AML much better than in solid tumors.

Finally, it is possible to hypothesize that, in the future, a triple combination of chemotherapy, DHODH inhibitors and dipyridamole. In this context, finding the correct schedule of administration will be fundamental to maximize the synergism and the pyrimidine depletion, while minimizing toxicity. A triple combination, especially if given continuously, could be toxic even for non-cancer cells; moreover, the block of nucleotide transporters could prevent some chemotherapeutic drugs from entering the cells, such as decitabine or Ara-C. However, a wise schedule of administration could further enhance the antileukemic activity and overcome chemo-resistance. Just to mention few examples, a pre-treatment with DHODH inhibitors and dipyridamole could (i) reduce the cytidine triphosphate (CTP) pool, sensitizing leukemic cells to azacytidine and decitabine by decreasing the competition for their incorporation into nucleic acids; and (ii) force leukemic cells to increase the expression of hENT channels, potentially promoting the access of decitabine, azacytidine or Ara-C into the cells.

## 4. Materials and Methods

### 4.1. Reagents

MEDS433 and brequinar were synthesized as described in [4]. ATRA, Teriflunomide, Dilazep and uridine were purchased from Sigma-Aldrich. Reagents were dissolved in DMSO and diluted in culture medium before use. Final DMSO concentration did not exceed 0.1%. Dilazep was dissolved in water. Ara-C (Citarabina Hospira, Lake Forest, IL, USA), Idarubicin (Zavedos, Pfizer, New York, NY, USA), Dipyridamole (Persantin, Boehringer Ingelheim, Ingelheim am Rheim, Germany), Decitabine (Dacogen, Janssen-Cilag, Beerse, Belgium) were purchased.

### 4.2. Cell Culture

All cell lines were purchased from DSMZ. The human cell lines THP1 (acute monocytic leukemia M5), U937 (pro-monocytic myeloid leukemia), NB4 (promyelocytic leukemia M3), MV4-11 (acute monocytic leukemia M5) and OCI AML3 (acute myelomonocytic leukemia M4) were maintained in RPMI 1640 (Gibco, Thermo Fisher Scientific, Waltham, MA, USA) supplemented with 10% heat-inactivated fetal bovine serum (FBS, Gibco, Thermo Fisher Scientific). The human stromal cell line HS-5 was cultured in DMEM (Microtech, Naples, Italy) supplemented with 10% FBS. Media were supplemented with 1% penicillin/streptomycin (Gibco, Thermo Fisher Scientific) and all cultures were maintained at 37 °C, 5% CO_2_. Cell lines were maintained in culture for no longer than 5–6 weeks and were routinely tested for mycoplasma contamination. The genetic alterations of cell lines are shown in Table S1. The doubling time of the cell lines was available in the DSMZ catalogue.

All cells lines were plated into 96-well round bottom plates at 1 × 10^4^ per well and treated with.

DMSO or a DHODH inhibitor: MEDS433 from 0.1 to 10 µM, brequinar from 0.1 to 10 µM or Teriflunomide from 50 to 100 µM for three days. Some experiments were repeated in the presence of uridine (concentration range: 1 to 100 µM). For drug combinations, cell lines were treated with ATRA 0.1 µM or Ara-C 1 µM or Idarubicin 0.005 µM or Decitabine 0.250 µM or Dypiridamole (0.01–10 µM). All experiments were repeated at least three times.

For hypoxia, 1 × 10^4^ THP1 cells were seeded into 96-well round bottom plates in a volume of 200 µL of complete RPMI. Cell cultures were grown under hypoxic conditions using a Hypoxia Chamber (STEMCELL Technologies, Vancouver, BC, Canada) at 37 °C, 5% CO_2_ and 1% O_2_. MEDS433 was added after a 24-h adaptation period to the hypoxic conditions. Cells were treated under hypoxic conditions for three days. Analysis of viability and differentiation were performed at the end of cultures.

### 4.3. Co-Culture

Stromal cell monolayers were generated seeding 1 × 10^3^ HS-5 in 96-well flat bottom tissue culture plates using Iscove’s modified Dulbecco’s medium (IMDM, Microtech), supplemented with 10% FBS. A total of 1 × 10^4^ THP1 were seeded on the stromal cell monolayer after 24 h and subsequently treated with MEDS443 (1 µM). After three days, the viability of THP1 was evaluated by Annexin V/Propidium Iodide (PI) assay (Miltenyi Biotec, Bergisch Gladbach, Germany). Cells in suspension were collected and pooled with adherent cells treated with Trypsin/ Ethylenediaminetetraacetic acid (EDTA, Sigma Aldrich, Milan, Italy). Cells were incubated with AnnexinV-FITC and CD4 APC (Miltenyi Biotec) for 15 min in the dark at room temperature. After PI addition, cells were acquired on FACSVerse (BD-Biosciences, San Jose, CA, USA) and THP1 cells were isolated from stroma cells by expression of CD4.

### 4.4. Primary Cells

Primary AML cells were obtained from peripheral blood or bone marrow of patients hospitalized in the University Division of Hematology and Cell Therapy of A.O. Ordine Mauriziano, Turin, Italy, after informed consent. Sample collection and analyses were approved by the Ethics Committee of the A.O. Ordine Mauriziano (deliberation number 602). All patients gave their informed consent for the use of their biological material and for publications of the obtained results. All samples contained >75% blasts as detected by flow cytometry and the characteristics of patients are shown in Table S2. Fresh or frozen samples were used. Mononuclear cells were purified by Ficoll-Hypaque density gradient centrifugation (Sigma Aldrich), and PBMC were cultured in IMDM (Microtech, Naples Italy) supplemented with 20% FBS, insulin–transferrin–selenium (Gibco, Thermo Fisher Scientific, Fms-like tyrosine kinase 3 Ligand (FLT3L, 20 ng/mL Miltenyi Biotec), Stem Cell Factor (SCF, (20 ng/mL Miltenyi Biotec), and granulocyte colony stimulating factor (G-CSF, 100 ng/mL, Nivestim, Hospira).

Mononuclear cells were isolated by Ficoll-Hypaque gradient from a healthy donor after they gave their informed consent. PBMC were maintained in complete RPMI 1640 and treated directly with MEDS433 alone or with Dypiridamole (0.1–1 µM) or stimulated with 2% phytohemagglutinin (PHA, Gibco, Thermo Fisher Scientific), IL2 200U/mL (Peprotech, Inc., Rocky Hill NJ, USA), and IL7 10 ng/mL (Miltenyi Biotec) for 72 h. After stimulation, cells were washed and re-suspended in complete RPMI 1640 supplemented with fresh cytokines and exposed to MEDS433 (0.1 µM), Idarubicin (0.005 µM), Ara-C (1 µM) for three days. Analysis of viability was performed by AnnexinV assay. To evaluate the lasting effect of the drugs on viability, cells were washed and re-plated in medium supplemented with fresh cytokines and uridine 5 µM and cultured for additional 6 days.

### 4.5. Annexin V and Differentiation Assays

Apoptosis was examined by flow cytometry using an Annexin V-FITC Kit (Miltenyi Biotec), according to the manufacturer’s instructions. Alternatively, cell lines or primary cells were incubated with Annexin V-FITC and CD14 APC (BD-Biosciences,) or CD11c APC (Gibco Thermo Fisher Scientific) for 15 min at room temperature in the dark. After the PI incorporation, samples were acquired on FACSVerse and analyzed by Kaluza software version 1.2 (Beckman Coulter, Brea, CA, USA).

### 4.6. Flow Cytometry

PBMC samples were seeded (1 × 10^4^ cells/well) in complete medium with or without the indicated concentrations of compounds. After 72 h incubation, cells were washed with PBS and stained with CD45RA PE (BD-Biosciences), CD62L APC (Miltenyi Biotec), CD3 FITC (Miltenyi Biotec) at room temperature for 20 min. Data collection was done on FACSVerse and dead cells were excluded from the analysis, based on the use of PI (Sigma-Aldrich).

For the evaluation of hENT2 protein expression, cell lines or AML primary cells were incubated with a SLC29A2 polyclonal antibody (Thermo fisher Scientific) using a dilution of 1:25 for 20 min at room temperature after fixation and permeabilization. The secondary antibody (Cell signaling Technology USA, Danvers, MA, USA) was used with a dilution of 1:200 for 20 min. Results were expressed as ΔGmean, i.e., the net mean fluorescence intensity (MFI) difference between the cells stained with primary and secondary antibodies and cells stained with the secondary antibody only, in order to eliminate the background positivity. All data were processed with Kaluza software version 1.2 (Beckman Coulter).

### 4.7. Caspase Activity Assay

Cells were plated in 96-well plates at the density of 10 × 103 cells/well. Caspase cleaving activity was evaluated by Caspase-Glo^®^ 3/7 Assay System (Promega, Madison, WI, USA) following the manufacturer’s instructions.

### 4.8. Cell Lysis and Western Blot Assay

Western blot analysis was performed as previously described [44]. Briefly, cells were lysed in lysis buffer (150 mM NaCl, 1 mM EDTA, 50 mM Hepes (pH 7.5), 1% Triton X-100 and 10% glycerol). Protein lysates were resolved in 4–20% SDS-PAGE gels and transferred into nitrocellulose filters. Proteins were visualized with peroxidase-conjugated secondary antibodies and chemiluminescence reagent (#170-5060 Bio-Rad, Hercules, CA, USA). We utilized antibodies targeting Cleaved Caspase 3 (#9661, Cell signaling Technologies, Danvers, MA, USA), Vinculin (SAB4200080) and DHODH (D-6) (sc-166377), both purchased from Santa Cruz.

### 4.9. RNA Extraction and Gene Expression Analysis

RNA was extracted from cells using TRIzol as previously described [45]. An amount of 1 μg of total RNA was used for reverse transcription with iScript cDNA Synthesis Kit (Bio-Rad) according to the manufacturer’s protocol. Real-time PCR parameters were: cycle 1, 95 °C—3 min; cycle 2, 95 °C—15 s, 60 °C—30 s for 40 cycles. The p53 quantification specific assays with on-demand primer/probe kits (Hs01034249_m1 for p53) (Applied Biosystems, Foster City, CA, USA) were conducted according to the manufacturer’s instructions. The 2-ΔΔCT method was used to analyze the data. GUS was used to normalize the results (Hs00939627_m1 for GUS) (Applied Biosystems).

### 4.10. Cell Staining

THP1 and NB4 were treated with MEDS433 at 1 µM for 3 days. Cells were washed in PBS and cytospin preparations were made at 1000 rpm for 5 min (Elitech Group, Puteaux, France). All slides were air dried and then stained with Wright–Giemsa using the Aerospray Hematology (Elitech Group) according to the manufacturer’s instructions.

### 4.11. Trypan Blue Staining Assay

For each experiment, all cells lines (1 × 10^4^) were treated for 3 days to the indicated concentrations of drugs in a final volume of 200 µL. Control groups were maintained in the same culture condition with DMSO. The total number of viable cells was determined by trypan blue staining (Sigma-Aldrich).

### 4.12. Statistical Analysis

Statistical analysis was either performed using Prism software, version 5.0 (GraphPad Software, San Diego, CA, USA), as well as a Python script that relied on the open-source SciKit Learn library. All data are reported as means ± SD with at least three independent experiments. A two tail paired Student’s t-test was used to assess differences between two groups and *p* < 0.05 was considered significant. A two-tailed unpaired Student’s *t*-test was used to measure the statistical significance of p53 expression experiment. For multiple comparisons, one-way ANOVA tests were performed and combined with Tukey’s tests for post hoc analyses. Moreover, in this case, a *p*-value < 0.05 was considered significant. Due to the high complexity of the figures, in some panels the statistical significance is shown only for the most relevant biological comparisons.

The linear regression that correlates the doubling time of the cells lines with the apoptotic rate was performed through the SciKit Learn library, and the obtained segment represents a linear interpolation between the means of the observations.

## 5. Conclusions

AML is an aggressive disease which is still characterized by a dismal prognosis in too many patients. Its biology reveals a heterogenous background, with multiple clones competing for selective advantage, but with some common features: a differentiation block, a reduced apoptosis and a metabolic addiction.

In this work we present a new combination, based on deep pyrimidine starvation and characterized by differentiating and pro-apoptotic features, that was effective on all AML cell lines and the vast majority of primary AML cells, with very different genetic backgrounds.

To the best of our knowledge, this is the first study investigating this combination treatment in AML; more importantly, as it addresses a completely new pathway, this treatment could be further associated with classical antileukemic drugs, in order to find the best combination to treat AML.

## Supplementary Materials

The following are available online at https://www.mdpi.com/2072-6694/13/5/1003/s1. Figure S1: MEDS433 reduces cell viability in several AML cell lines and its activity is preserved in niche-like conditions. Figure S2: p53 expression in AML cell lines treated with MEDS433 for 1 day, as evaluated in real time PCR. Figure S3: Kinetic of apoptosis and differentiation of U937 cells treated with MEDS433 at 1 μM. Figure S4: The combination of MEDS433 with classical antileukemic agents results in near-additive. Figure S5: The combination of MEDS433 and hENT1/2 blockers induces apoptosis in AML cell lines, even when in vivo conditions are mimicked. Figure S6: Activity of MEDS433, dipyridamole and their combination at hyperphysiological uridine concentrations. Figure S7: Toxicity of MEDS433 and dipyridamole on monocytes. Figure S8: Original Western blot figures of Figure 2C. Figure S9: Original Western blot figures of Figure 3D. Table S1: Molecular alterations of utilized cell lines. Table S2: WHO classification and molecular alterations of utilized AML samples.

## Data and Sample Availability

Experimental data are available from the authors upon reasonable request and with the permission of the University of Turin.
