# Supplementary material for: The Synergism between DHODH Inhibitors and Dipyridamole Leads to Metabolic Lethality in Acute Myeloid Leukemia
Source: Cancers (Basel). 2021 Feb 28;13(5):1003. doi: 10.3390/cancers13051003 (PMC7957697; doi:10.3390/cancers13051003)
Supplement: Supplementary file 1 [file cancers-13-01003-s001.pdf]

Supplementary Material: The Synergism between DHODH Inhibitors and Dipyridamole Leads to Metabolic Lethality in Acute Myeloid Leukemia

Valentina Gaidano, Mohammad Houshmand, Nicoletta Vitale, Giovanna Carrà, Alessandro Morotti, Valerio Tenace, Stefania Rapelli, Stefano Sainas, Agnese Chiara Pippione, Marta Giorgis, Donatella Boschi, Marco Lucio Lolli, Daniela Cilloni, Alessandro Cignetti, Giuseppe Saglio and Paola Circosta

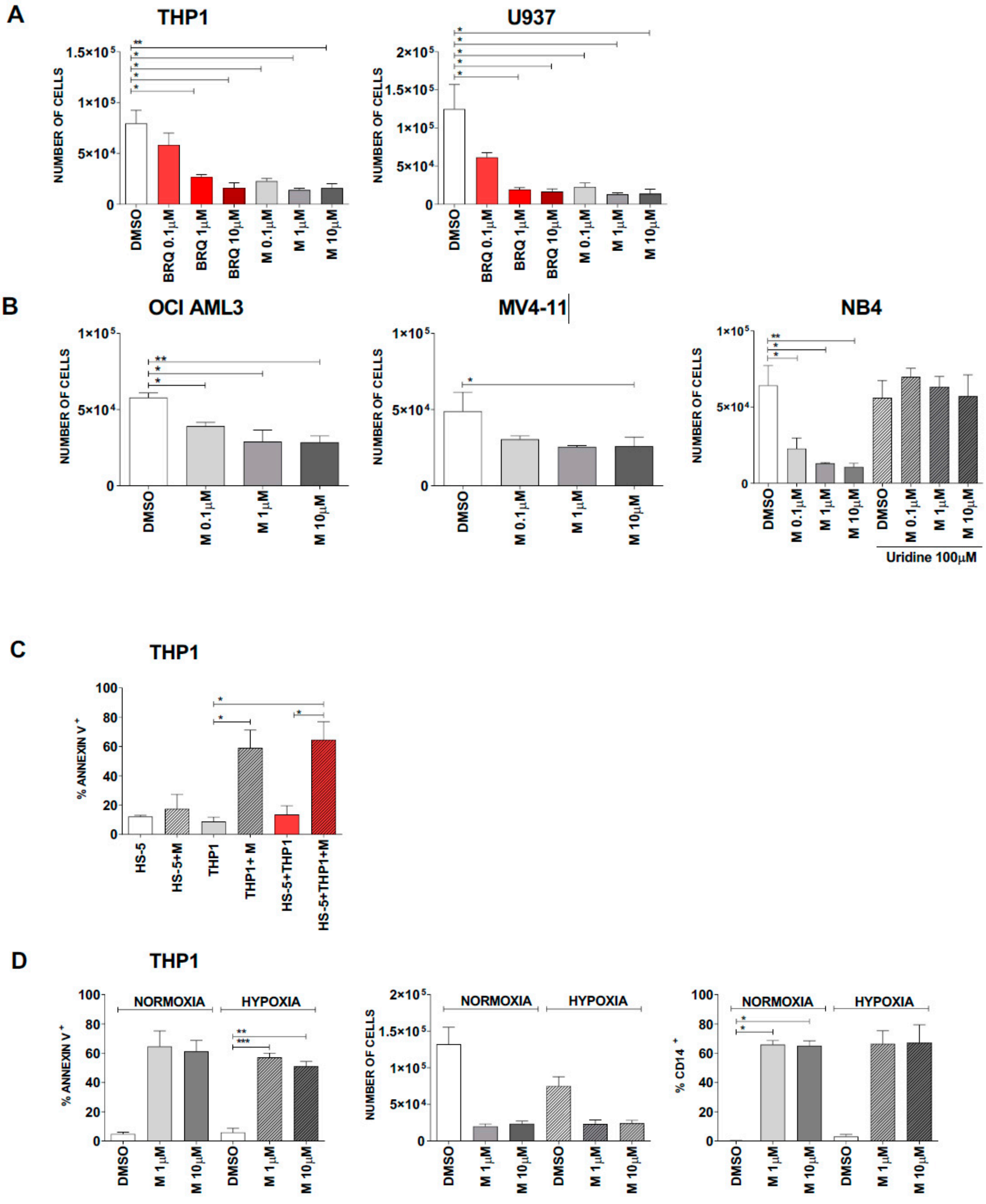

Figure S1. MEDS433 reduces cell viability in several AML cell lines and its activity is preserved in niche-like conditions (related to Figure 1). (**A**) Reduction of THP1 and U937 cell viability after treatment with MEDS433 or brequinar at increasing concentrations ($n = 3$). (**B**) Reduction of OCI AML3, MV4-11 and NB4 cell viability after treatment with MEDS433 at increasing concentrations ($n = 3$). Hyperphysiological uridine concentrations rescue cell viability (right panel, $n = 3$). (**C**)

and (**D**) MEDS433 activity is preserved in niche-like conditions. In (**C**), the apoptotic rate induced by MEDS433 at 1 μM is preserved when THP1 cells are co-cultured with HS-5, a stromal cell line (*n* = 3). (**D**) Apoptotic rate (left panel), number of viable cells (middle panel) and differentiation effect (right panel) induced by MEDS433 in normoxic and hypoxic conditions (*n* = 3). In all experiments, apoptosis and differentiation were evaluated after 3 days of treatment. Apoptosis was evaluated through Annexin V expression; cell differentiation was evaluated through CD14 expression. DMSO indicates cells treated with dimethyl sulfoxide only. M: MEDS433. BRQ: brequinar. Ur: uridine. Statistical significance: *t*-test, *$p < 0.05$; **$p < 0.01$; ***$p < 0.001$.

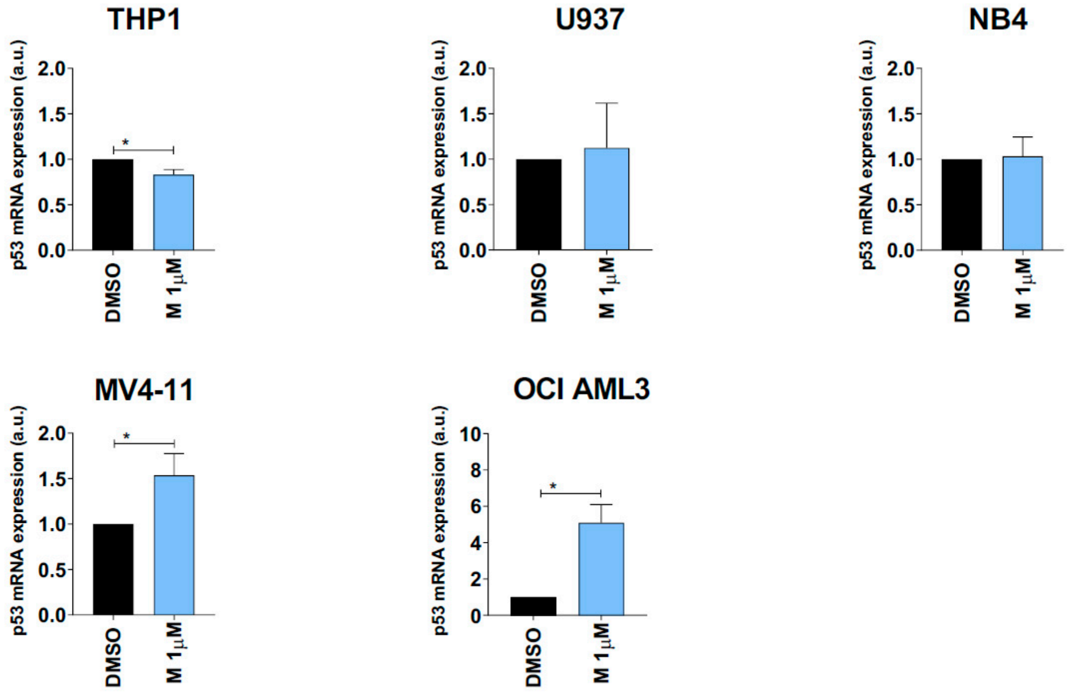

Figure S2. p53 expression in AML cell lines treated with MEDS433 for 1 day, as evaluated in real time PCR (*n* = 3) (related to Figure 1). Measurements are normalized to the expression levels of untreated cells. DMSO: dimethyl sulfoxide. M: MEDS433. Statistical significance: unpaired *t*-test, *$p < 0.05$; **$p < 0.01$; ***$p < 0.001$.

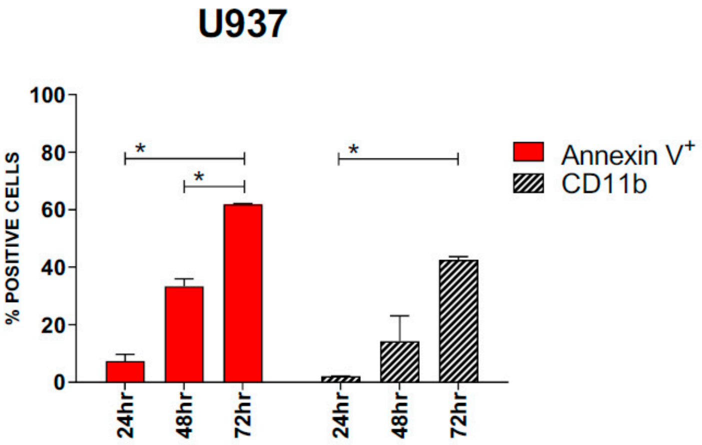

Figure S3. Kinetic of apoptosis and differentiation of U937 cells treated with MEDS433 at 1 μM (*n* = 3) (related to Figure 4).. Statistical significance: *t*-test, *$p < 0.05$;.

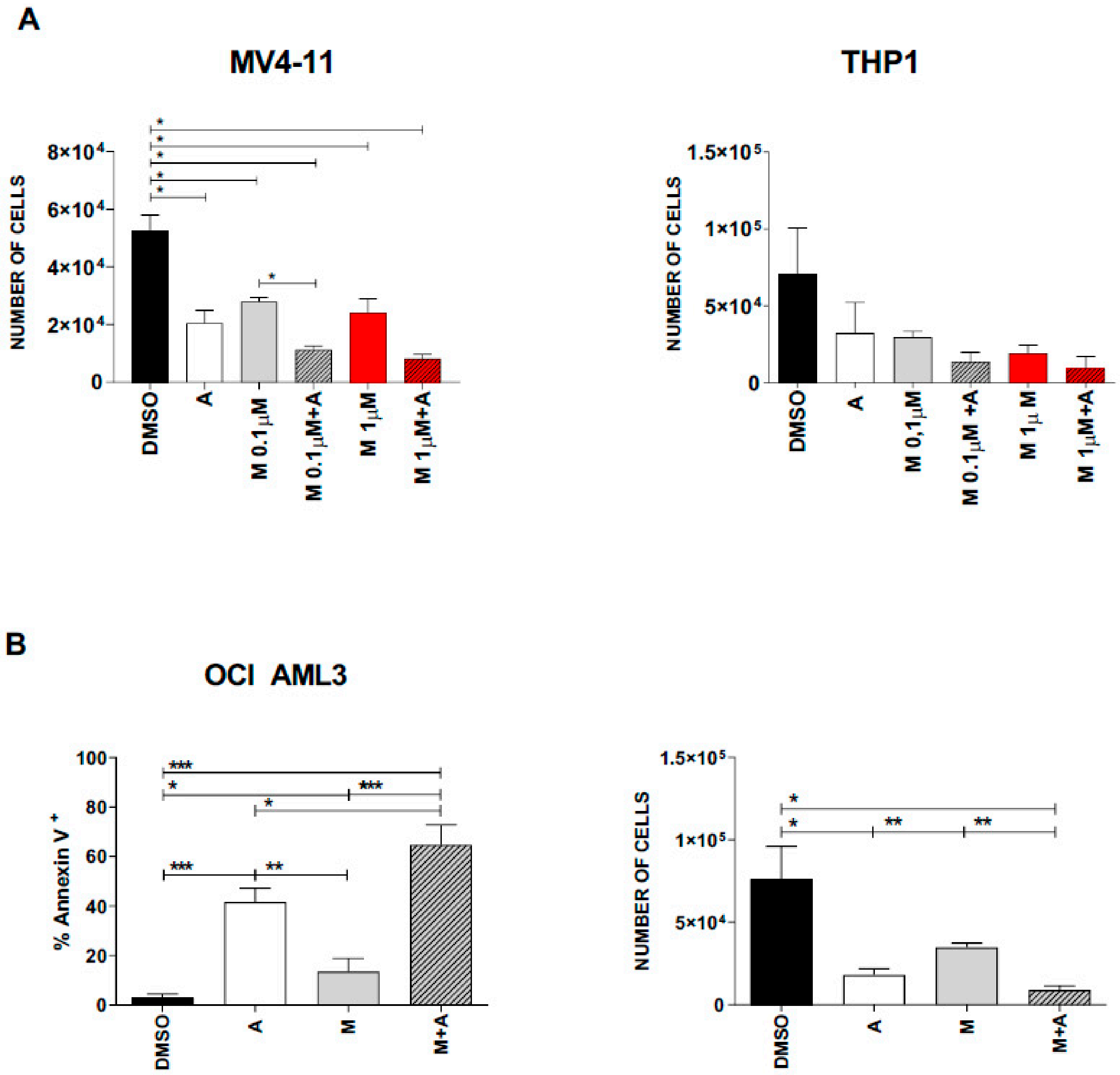

Figure S4. The combination of MEDS433 with classical antileukemic agents results in near-additive (related to Figure 5). (**A**) MV4-11 and THP1 cells viability after treatment with MEDS433, Ara-C and their combination (n = 3). (**B**) Apoptotic rate (left panel) and cell viability (right panel) of OCI AML3 cells treated with MEDS433 1μM, Ara-C or both (n = 3). Analyses were performed after 3 days of treatment. DMSO: dimethyl sulfoxide. M: MEDS433. A: Ara-C. Statistical significance: Anova/Tukey, *$p < 0.05$; **$p < 0.01$; ***$p < 0.001$; ****$p < 0.0001$.

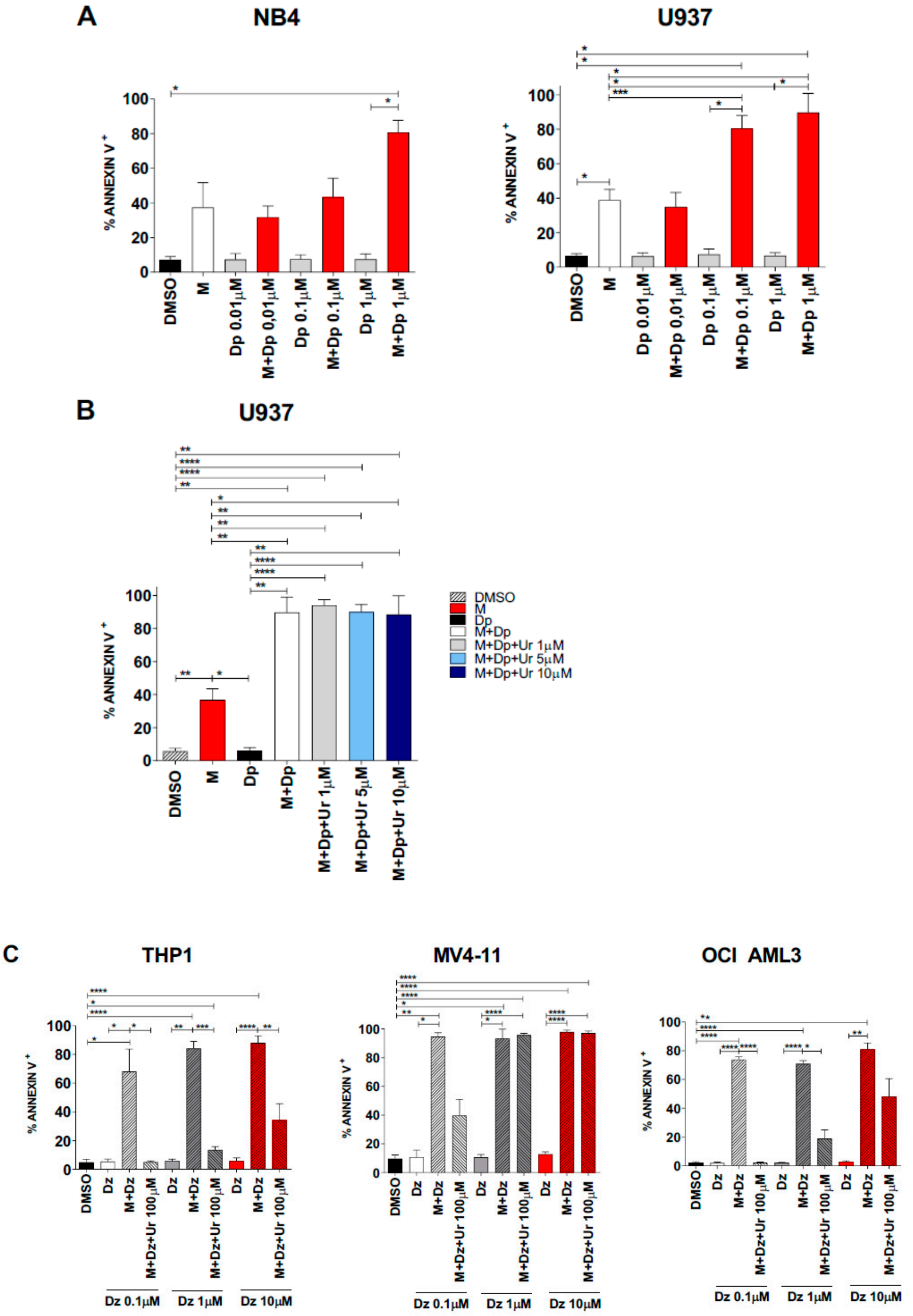

Figure S5. The combination of MEDS433 and hENT1/2 blockers induces apoptosis in AML cell lines, even when in vivo conditions are mimicked (related to Figures 6 and 7). (**A**) Apoptosis induced by MEDS433, dipyridamole and their combination on NB4 and U937 cells (*n* = 3). (**B**) Apoptosis induced by MEDS433 in combination with dipyridamole in the presence of uridine at low concentrations (1 to 10 µM) on U937 cells (*n* = 3). (**C**) Apoptosis induced by MEDS433, dilazep or their combination, with and without uridine at 100 µM, on THP1 (*n* = 4), MV4-11 (*n* = 3) and OCI AML3 (*n* = 3)cells. Dilazep was utilized at increasing concentrations, as shown in the figure. DMSO: dimethyl sulfoxide. M: MEDS433. Dp: dipyridamole. Dz: dilazep. Ur: uridine. Apoptosis was evaluated after 3 days of treatment. Statistical significance: Anova/Tukey, *$p < 0.05$; **$p < 0.01$; ***$p < 0.001$; ****$p < 0.0001$.

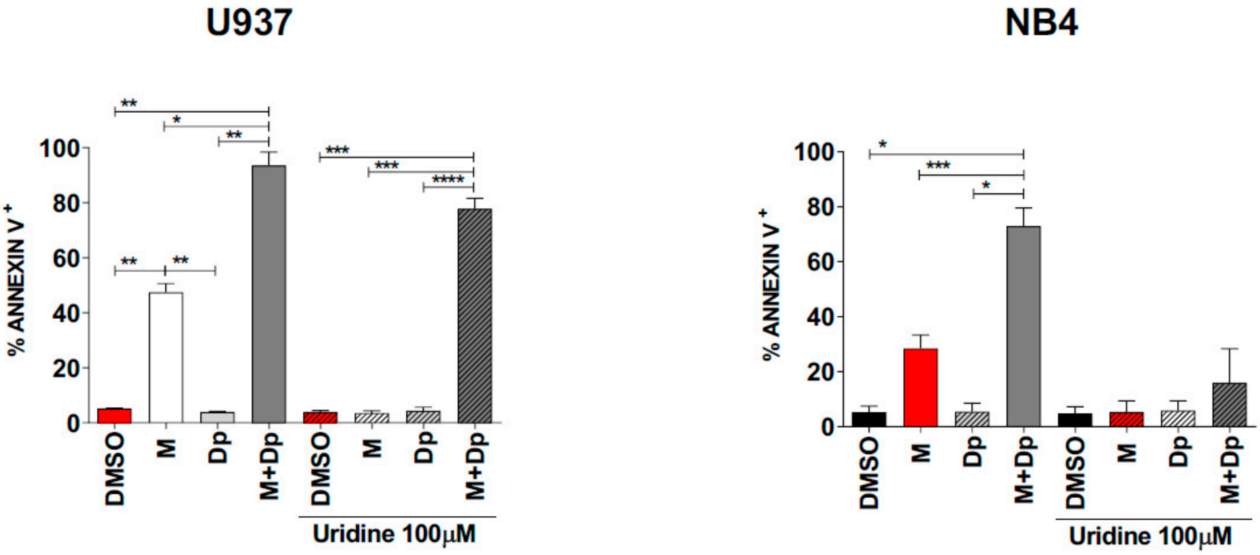

Figure S6. Activity of MEDS433, dipyridamole and their combination at hyperphysiological uridine concentrations (related to Figure 8). Apoptosis induced by MEDS433, dipyridamole or their combination, with and without uridine at 100 μM, on U937 (*n* = 3) and NB4 cells (*n* = 3). In all experiments, MEDS433 was utilized at 0.1 μM, dipyridamole at 1 μM and apoptosis was evaluated after 3 days of treatment. DMSO: dimethyl sulfoxide. M: MEDS433. Dp: dipyridamole. Ur: uridine. Statistical significance: Anova/Tukey, *$p < 0.05$; **$p < 0.01$; ***$p < 0.001$; ****$p < 0.0001$.

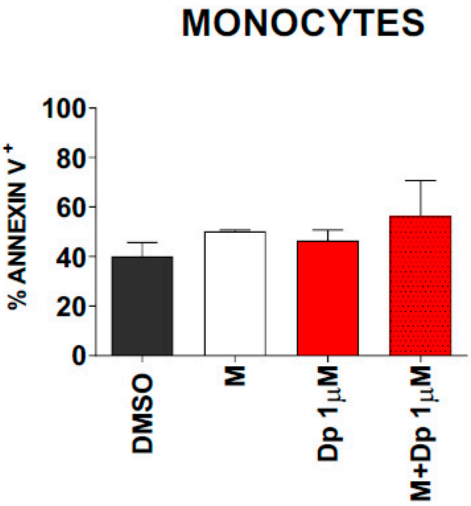

Figure S7. Toxicity of MEDS433 and dipyridamole on monocytes (related to Figure 10). Apoptosis of monocytes treated with MEDS433 alone (0.1 μM) or in combination with dipyridamole (1 μM). Apoptosis was evaluated after 3 days of treatment (*n* = 3). DMSO: dimethyl sulfoxide. M: MEDS433. Dp: dipyridamole. Statistical significance: Anova/Tukey, *$p <$ 0.05; **$p < 0.01$; ***$p < 0.001$; ****$p < 0.0001$.

Table S1. Molecular alterations of utilized cell lines. All cell lines are characterized by complex karyotypes.

Cell Line	Genetic Alterations
U937	CALM-AF10, PTEN, TP53

THP1	NRAS, TP53, MLL-AF9, CSNK2A1-DDX39B
OCI AML3	DNMT3A, NPM
NB4	PML-RARa, KRAS, TP53
MV4-11	MLL-AFF1, FLT3-ITD

Table S2. WHO classification and molecular alterations of utilized AML samples.

PT	Type of Sample	WHO Classification-Genetic Alterations
1	PB	AML with myelodysplasia-related changes
2	PB	AML with myelodysplasia-related changes
3	BM	Therapy-related myeloid neoplasms
4	BM	AML NOS, without maturation
5	PB	AML NOS, Acute myelomonocytic leukemia
6	BM	AML NOS, Acute myelomonocytic leukemia (FLT3+)
7	PB	AML NOS, without maturation (secondary to MPN, myeloproliferative neoplasm)
8	PB	Provisional entity: AML with BCR-ABL1 (blast crisis of CML)
9	PB	AML NOS, Acute myelomonocytic leukemia (FLT3+)
10	PB	AML NOS, Acute myelomonocytic leukemia (FLT3+
11	PB	AML NOS, without maturation (secondary to MPN, myeloproliferative neoplasm)
12	PB	AML NOS, Acute myelomonocytic leukemia (FLT3+)

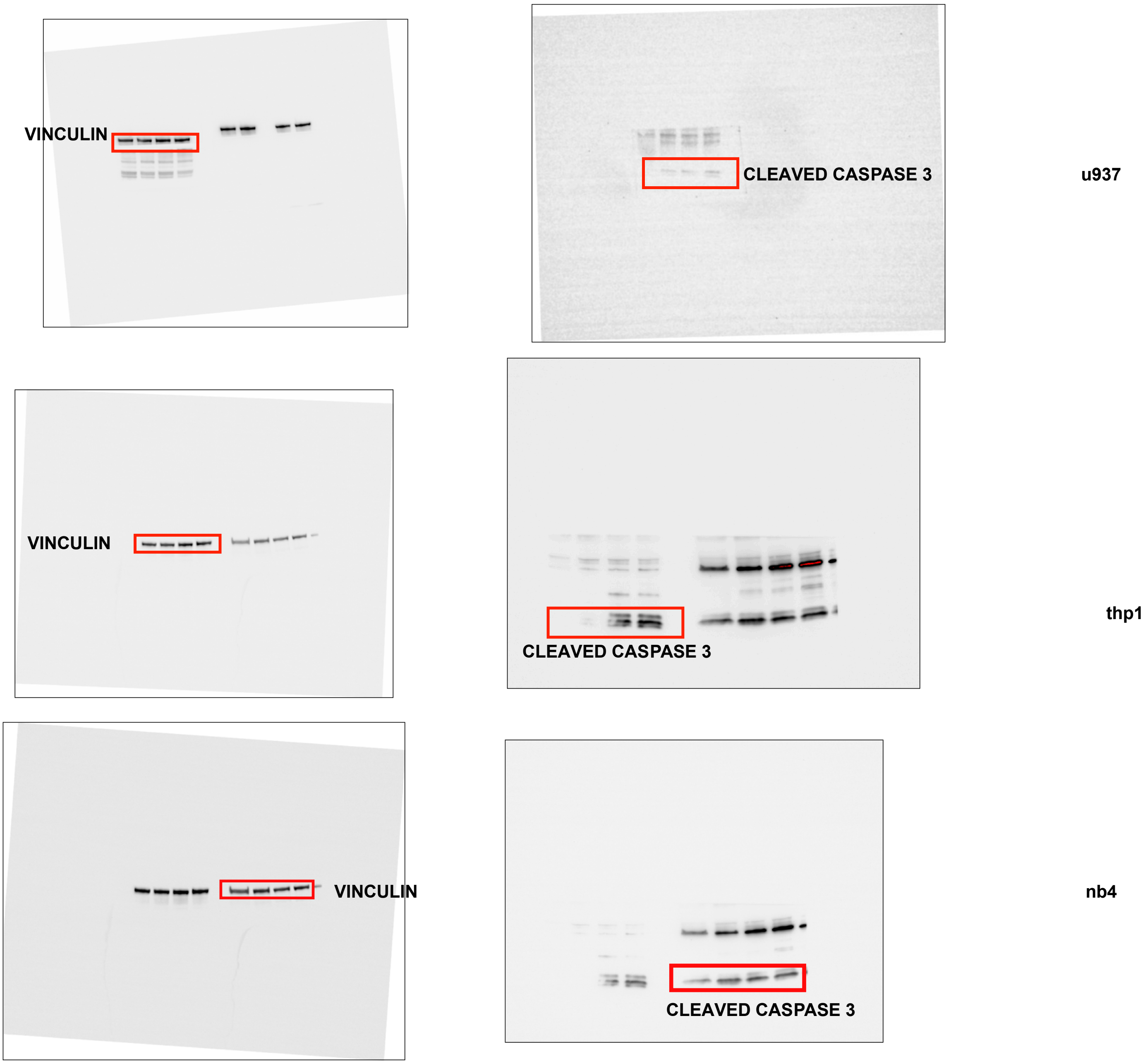

Figure S8. Original Western blot figures of Figure 2B.

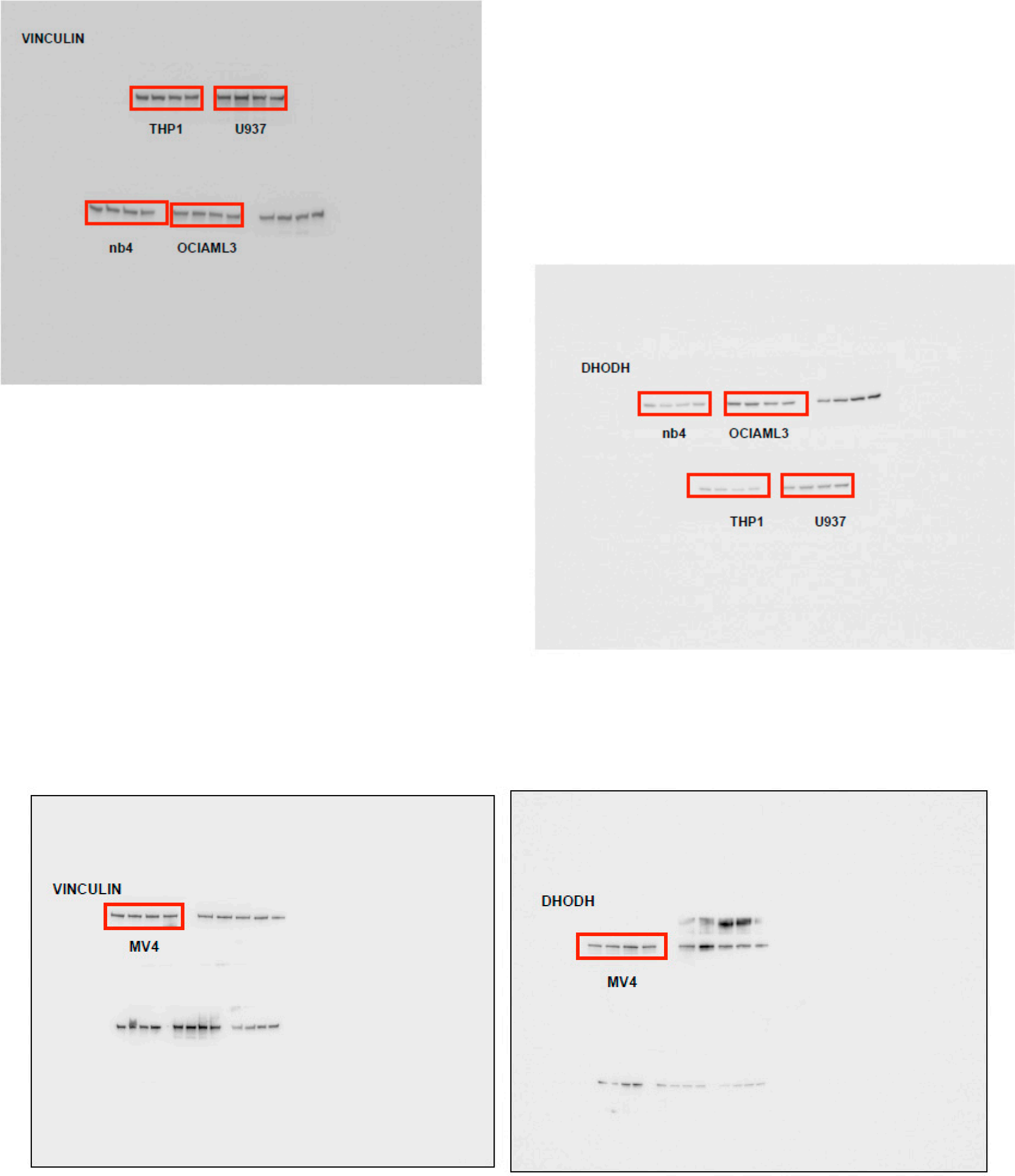

Figure S9. Original Western blot figures of Figure 3D.
